# Response To Letter To The Editor Regarding Article "Repolarization Parameters Are Associated With Mortality In Chagas Disease Patients In The United States"

**DOI:** 10.1016/s0972-6292(16)30822-1

**Published:** 2014-12-15

**Authors:** J Bradfield, B Woodbury, M Traina, S Hernandez, D Sanchez, R Wachsner, K Shivkumar, S Meymandi

**Affiliations:** UCLA Cardiac Arrhythmia Center, UCLA Health System, David Geffen School of Medicine at UCLA, Los Angeles, CA and Center of Excellence for Chagas Disease, Olive View-UCLA Medical Center, Sylmar, CA

**Keywords:** Fragmented QRS, Chagas' disease

We thank Dr. Baranchuk and colleagues for their thoughtful and insightful comments regarding our manuscript.

While the primary focus of the manuscript was repolarization abnormalities we regretfully did not cite the authors important work on ECG fragmentation. Unfortunately, the authors' abstract [1,2] was not noted in our initial literature search. The authors subsequently reported their data in manuscript form [3] in January of 2014. This should serve as a reminder to consider a repeat literature review after an initial manuscript is submitted, as failing to do so can lead to an incomplete review when new data becomes available in the interim.

An important point is raised regarding the lack of association seen between fragmentation and appropriate shocks in the FECHA study. [1,3] Fragmentation may be an indicator of scar presence, but perhaps not directly related to arrhythmia risk. The FECHA study provides important insight, but should be interpreted in context, as the patient population studied was high risk (70% secondary prevention indication for ICD). Therefore, fragmentation may not be expected to be an independent risk factor for appropriate ICD therapy given the appropriate therapy rates in a secondary prevention population. Further, the increased use of beta-blockers in the fragmented QRS group may have decreased the differences between groups.

In our manuscript we found increased fragmentation in the Chagas Disease patients with cardiomyopathy relative to their non-ischemic cardiomyopathy counterparts, as well as increased fragmentation in Chagas Disease patients with normal ejection fraction, but conduction system involvement, when compared to controls. Fragmentation was not a predictor of death or transplant as a univariate variable in our study. Fragmentation in our patient population will be reassessed in a larger cohort with MRI correlation in future studies.

We believe that scar burden should predict severity of disease and arrhythmia risk, and there is precedence for this in the Chagas Disease population. [4-6] However, patients with non-ischemic cardiomyopathy, while often having delayed enhancement on cardiac MRI and low voltage on electroanatomic mapping during electrophysiologic study, tend to have arrhythmia burden out of proportion to the burden of documented scar. This may be due to microfibrosis as the authors point out. Another potential explanation may be the occurrence of functional ventricular arrhythmias in this population, as opposed to reentrant arrhythmias involving large scars described in an ischemic cardiomyopathy population. The involvement of the autonomic nervous system, and the destruction of said system, in Chagas Disease may also be a potential contributing factor to the increased ventricular arrhythmia risk in this patient population and requires further study.
